# Estrategia educativa para la prevención del cáncer de cuello uterino en indígenas: una experiencia de investigación-acción participativa

**DOI:** 10.1590/0102-311XES170423

**Published:** 2025-04-25

**Authors:** Sandra Lucía Vargas-Cruz, Claudia Marcela Velásquez-Jiménez, Vilma Fandiño-Osorio, María Sarmiento-Medina, María Monsalve-Córdoba, Miryam Puerto-De Amaya

**Affiliations:** 1 Universidad El Bosque, Bogotá, Colombia.; 2 Universidad de Ciencias Aplicadas y Ambientales, Bogotá, Colombia.; 3 Vicerrectoría de Investigaciones, Fundación Universitaria de Ciencias de la Salud, Bogotá, Colombia.

**Keywords:** Educación en Salud, Neoplasias del Cuello Uterino, Participación de la Comunidad, Pueblos Indígenas, Health Education, Uterine Cervical Neoplasms, Community Participation, Indigenous Peoples, Educação em Saúde, Neoplasias do Colo do Útero, Participação da Comunidade, Povos Indígenas

## Abstract

El objetivo fue evaluar los cambios en los conocimientos, actitudes y prácticas sobre el cáncer de cuello uterino después de una intervención educativa en el marco de investigación-acción participativa con pueblos indígenas del Resguardo El Paujil, Guainía, Colombia. Se realizó un diagnóstico con enfoque intercultural y participativo. Posteriormente, se diseñó e implementó la estrategia educativa. La investigación en todas sus etapas tuvo participación de lideresas de la comunidad. La estrategia se evaluó mediante una encuesta sobre conocimientos, actitudes y prácticas sobre el cáncer de cuello uterino. Los efectos se determinaron al comparar los cuestionarios antes y después de la intervención con la prueba de Mann-Whitney y el chi-cuadrado. Participaron en la estrategia educativa 957 mujeres. Hubo un aumento significativo en el nivel de conocimientos sobre el cáncer de cuello uterino antes (8,5%) y después (12,5%) de la intervención (p < 0,001) y en la práctica de la citología vaginal (64,4% y 73,9%; p = 0,0467). Sin embargo, el escaso conocimiento que tenían sobre la relación del virus del papiloma humano con el cáncer de cuello uterino y sobre factores de riesgo como la multiparidad o el inicio temprano de relaciones sexuales no cambió con la intervención. Se encontró un aumento significativo en los conocimientos de las mujeres de bajo nivel educativo después de la intervención. La educación en salud con enfoque intercultural en el marco de la investigación-acción participativa es efectiva para mejorar los conocimientos y prácticas para la prevención del cáncer de cuello uterino en comunidades vulnerables. Sin embargo, para obtener mejores resultados es importante que los proyectos sean de mayor duración para construir relaciones de confianza con las comunidades.

## Abstract (en)

This study aimed to evaluate changes in knowledge, attitudes, and practices on cervical cancer after an educational intervention in the framework of participatory action research with Indigenous peoples of the El Paujil reservation, Guainía, Colombia. A diagnosis was made with an intercultural and participatory approach. Subsequently, the educational strategy was designed and implemented. Community leaders actively participated in the research during all stages. The strategy was evaluated via a survey on knowledge, attitudes, and practices about cervical cancer. The effects were determined by comparing pre- and post-intervention questionnaires with the Mann-Whitney and chi-squared tests. Overall, 957 women participated in the educational strategy. The level of knowledge about cervical cancer significantly improved, increasing from 8.5% to 12.5%, before and after the intervention (p < 0.001), respectively. The percentage of Pap smear testing also increased, from 64.4% to 73.9% (p = 0.0467). However, the limited knowledge about the relationship between HPV and cervical cancer, and about risk factors such as multiparity or early initiation of sexual intercourse remained the same. After the intervention, a significant increase in the knowledge of women with low educational level was found. Health education with an intercultural approach within the participatory action research framework is effective in improving knowledge and practices for the prevention of cervical cancer in vulnerable communities. However, for optimal results, it is important to hold long-term projects in order to build trust relationships with the communities.

## Abstract (pt)

Este estudo buscou identificar as mudanças de conhecimento, atitudes e práticas sobre o câncer do colo do útero após uma intervenção educativa de uma pesquisa-ação participativa com povos indígenas da Reserva El Paujil, Guainía, na Colômbia. Foi realizado um diagnóstico a partir de uma abordagem intercultural e participativa. Posteriormente, foi elaborada e implementada uma estratégia educacional. A pesquisa contou com a participação de lideranças comunitárias em todas as suas etapas. A estratégia foi avaliada por meio de um questionário sobre conhecimentos, atitudes e práticas sobre o câncer do colo do útero . Os efeitos foram determinados pela comparação dos questionários pré e pós-intervenção com o teste de Mann-Whitney e qui-quadrado. Participaram 957 mulheres da estratégia educacional. Houve aumento significativo em relação ao nível de conhecimento sobre câncer do colo do útero (de 8,5% antes para 12,5% após a intervenção; p < 0,001) e na realização do exame Papanicolau (de 64,4% para 73,9%; p = 0,0467). No entanto, o conhecimento limitado das participantes sobre a relação do HPV com o câncer do colo do útero e sobre fatores de risco como multiparidade ou início precoce da vida sexual não se alterou com a intervenção. Houve um aumento significativo do conhecimento das mulheres com baixa escolaridade após a intervenção. A educação em saúde centrada em uma abordagem intercultural no âmbito da pesquisa-ação interpretativa é eficaz para melhorar os conhecimentos e práticas de prevenção do câncer do colo do útero em comunidades vulneráveis. No entanto, para obter melhores resultados, é importante que os projetos tenham uma duração mais longa para criar relações de confiança com as comunidades.

## Introducción

El cáncer de cuello uterino es el cuarto cáncer más frecuente en la mujer, con una incidencia estimada de 604.000 nuevos casos en 2020. De las 342.000 muertes estimadas por cáncer de cuello uterino en 2020, más del 90% ocurrieron en países de ingresos bajos y medianos ^1^ ^,^ ^2^. En Latinoamérica y el Caribe es el segundo cáncer más frecuente, ocasionando una mortalidad de aproximadamente 35.000 mujeres cada año ^3^.

Aunque el cáncer de cuello uterino es prevenible y curable cuando se detecta a tiempo y se trata adecuadamente ^4^, sigue afectando de manera importante a las mujeres pertenecientes a poblaciones vulnerables como las indígenas ^5^ ^,^ ^6^. Específicamente para este proyecto las indígenas de Guainía en Colombia, permanentemente están enfrentando situaciones de pobreza, falta de conocimiento sobre enfermedades prevenibles como el cáncer de cuello uterino y dificultades en el acceso a las acciones de prevención como la vacunación, el tamizaje y el tratamiento oportuno ^7^.

El tamizaje sigue siendo una de las mejores alternativas para la detección temprana de lesiones y actualmente se ha avanzado en el desarrollo de nuevas técnicas y pruebas más sensibles y específicas para el virus del papiloma humano (VPH), tales como el método de citología en base líquida, la lectura automatizada, la identificación del ADN viral que permiten detectar la población en riesgo para aplicar sobre ellas estrategias de diagnóstico y tratamiento oportuno de lesiones precursoras del cáncer de cuello uterino ^8^.

La atención primaria en salud (APS), componente fundamental del sistema de salud, tiene un papel central en el control del cáncer de cuello uterino con la realización de las pruebas de tamizaje, la movilización comunitaria, la educación sanitaria y la generación de una alta cobertura de vacunación, detección y adherencia al tratamiento ^9^. Sin embargo, los países sudamericanos enfrentan desafíos para consolidar modelos de atención basados ​​en una APS integral ^10^, por lo que la carga de enfermedad asociada con cáncer de cuello uterino es alta, sin cambios significativos en las tasas de mortalidad a pesar de la reducción de la incidencia en algunos países ^11^.

En ese contexto, la educación para la salud se convierte en una de las estrategias más importantes en la prevención del cáncer de cuello uterino y exige de los profesionales de salud no solo competencias comunicativas, sino interculturales y sociales que les permitan un verdadero diálogo de saberes. El valor agregado para las comunidades, es el de tener información clara sobre los recursos que están disponibles y los beneficios de acceder a esos servicios, sobre todo para las enfermedades prevenibles o de tratamiento precoz, como lo es el cáncer de cuello uterino. La evidencia ha mostrado que diferentes intervenciones en salud con una mirada Intercultural y con estrategias acordes a los contextos, son efectivas porque permiten reflexión sobre los comportamientos propios y tomar decisiones informadas sobre el cuidado de uno mismo, de las familias y de las comunidades ^12^ ^,^ ^13^.

La investigación-acción participativa (IAP) se convierte en una oportunidad para que los equipos de salud realicen autorreflexión sobre sus prácticas medicalizadas y generen estrategias educativas y de comunicación no para las comunidades, sino con ellas mismas. La IAP, surgió en América Latina con el liderazgo de Orlando Fals Borda con dos ideas fundantes: valorar y respetar el conocimiento popular y, por otro, articular la Ciencia de la academia clásica con la tradición popular, de tal manera que, al integrar la rigurosidad científica con la acción y la participación, se materialice la transformación de la realidad ^14^. De esta manera, en América Latina se han realizado múltiples estudios con el enfoque de IAP, en diferentes campos como la educación, la salud pública y comunitaria, las Ciencias Sociales y el desarrollo comunitario ^15^. En algunos casos, los proyectos de IAP en temas de salud comunitaria han llevado a la implementación de programas de salud más inclusivos y basados en las necesidades reales de las comunidades, tomando en cuenta sus aspectos culturales ^16^ ^,^ ^17^. Además, se generan procesos de los colectivos mediante diálogos interculturales que les permiten llegar a acuerdos para acceder a las tecnologías actuales que la evidencia científica ha declarado como las mejores para el cuidado y mantenimiento de la salud y de esta manera contribuir al logro de políticas y programas estatales establecidos ^18^ ^,^ ^19^. En la población indígena latinoamericana se han realizado múltiples investigaciones en salud con el enfoque IAP en diferentes países como México, Brasil, Ecuador y Colombia. Estos estudios abordan diversos temas como la salud materno infantil, alimentación y nutrición y modelos de atención en salud con enfoque intercultural, entre otros ^16^ ^,^ ^17^ ^,^ ^20^ ^,^ ^21^ ^,^ ^22^.

El objetivo de este estudio fue establecer si la estrategia de comunicación y educación sobre el cáncer de cuello uterino construida en el marco de investigación acción participativa mejoró los conocimientos, actitudes y prácticas relacionadas con esta enfermedad en mujeres indígenas pertenecientes a las etnias mayoritarias del Resguardo El Paujil en Guainía, Colombia.

## Metodología

El estudio se llevó a cabo en el Resguardo El Paujil ubicado en la zona periurbana de Puerto Inírida, Guainía, al suroriente de Colombia. Este resguardo tiene una población aproximada de 4.000 habitantes pertenecientes a comunidades indígenas y no indígenas nacionales y extranjeros. A pesar de la gran influencia del mundo occidental por su cercanía el área urbana, aún conservan actividades propias de los pueblos indígenas y las viviendas son construidas de manera tradicional y no cuentan con un sistema de alcantarillado y saneamiento básico adecuado. Las actividades laborales principales son la ganadería, la pesca y la agricultura. Además, su territorio se ha visto afectado por la explotación minera legal e ilegal y la presencia de grupos armados ilegales ^23^ ^,^ ^24^. Se incluyeron cinco de las 13 etnias del resguardo por ser las de mayor tamaño: Puinave, Piapoco, Curripaco, Sikuani y Cubeo. Se incluyeron mujeres indígenas mayores de 18 años o menores de 18 años si ya tenían pareja.

Este estudio se realizó en el marco de la metodología de IAP, entendida como un método de identificación, análisis y aprendizaje colectivo de la realidad propia con la participación activa de los grupos implicados, que tiene como propósito estimular acciones y estrategias para transformarla y mejorarla.

El método de IAP combina tres procesos: (a) la investigación como procedimiento reflexivo, sistemático, controlado y crítico que tiene por finalidad estudiar algún aspecto de la realidad con una expresa finalidad práctica; (b) la acción reflexionada que representa una fuente de conocimiento, al tiempo que una forma de intervención para la transformación de la realidad; y (c) la participación como el proceso transversal en el que está involucrada la comunidad destinataria del proyecto. La comunidad no es considerada como objeto de investigación, sino como sujeto activo que contribuye a conocer y transformar su realidad en un ejercicio de interculturalidad.

### Fase de investigación

Diagnóstico e inmersión en las comunidades, protagonizada por cinco lideresas indígenas. Inicialmente, se realizó con ellas una capacitación basada en el diálogo de saberes y desde el enfoque de la interculturalidad crítica que incluyó temas prácticos de recolección de información cualitativa, manejo de tecnologías para archivo de información, prevención del cáncer de cuello uterino, liderazgo y ética de la investigación. Las lideresas se desplazaron a las casas de las mujeres del resguardo indígena para hacer entrevistas en lenguas ancestrales que fueron grabadas y trascriptas.

La guía de entrevista semiestructurada se realizó de manera conjunta con las lideresas, respetando las costumbres de las mujeres indígenas de acuerdo con lo que las lideresas conocían sus propias culturas, lo que permitió indagar sobre actitudes hacia el cuidado de la salud.

Con base en este diagnóstico, se concluyó que había una actitud positiva hacia los programas de salud siempre y cuando tuvieran en cuenta las diferencias culturales y que la propuesta de tamizaje de cáncer de cuello uterino no entraba en conflicto con su cosmovisión del proceso salud-enfermedad.

### Fase de acción

#### Diseño de la estrategia educativa

A partir de la información recolectada, se procedió al diseño de varios materiales educativos y estrategias informativas sobre el cáncer de cuello uterino en forma conjunta entre el equipo de investigadoras y las lideresas. Producto de ello se elaboraron cartillas, volantes, afiches, mensajes para perifoneo y redes sociales. Todos los materiales fueron aprobados por las autoridades tradicionales y traducidos a las lenguas indígenas por los ancianos conocedores de la lengua.

Se exploraron varios enfoques educativos con comunidades indígenas, intentando encontrar estrategias de doble vía, que permitieran el diálogo intercultural y evitaran al máximo una posición epistemológica dominante. Por lo cual se seleccionó el constructivismo por ser una estrategia pedagógica más flexible para llevar a cabo procesos interculturales y participativos ^19^ ^,^ ^25^. Las estrategias educativas fueron propuestas por las lideresas.

#### Aplicación de la estrategia educativa

Se llevó a cabo a través de varias metodologías.

(i) Visitas casa a casa por parte de las lideresas quienes fueron capacitadas previamente para informar personalmente a las mujeres en su lengua autóctona sobre el cáncer de cuello uterino mediante un rotafolio. Se entregaron las cartillas y se invitó a la toma de citologías y pruebas de VPH. Al comienzo de la visita, se aplicó la encuesta sobre conocimientos, actitudes y prácticas relacionados con cáncer de cuello uterino.

(ii) Las visitas se complementaron con mensajes radiales en lenguas ancestrales y en español, informando sobre la importancia de la prevención del cáncer de cuello uterino, afiches distribuidos en las tiendas, casas comunales y sitos de afluencia de personas. También se realizó un programa de radio de 30 minutos sobre el tema, divulgación desde el hospital, el puesto de salud y las instituciones de salud. Se realizó difusión local mediante perifoneo, mensajes en redes sociales, entrega de volantes y en reuniones comunitarias. Se hizo también presentación de la estrategia y de las campañas de citología en otras instituciones para que divulgaran la información.

#### Tamizaje de cáncer cervicouterino

Se realizaron tres jornadas de tamizaje en el centro de salud del resguardo, en las cuales, previa a la toma de la citología, se les informó en sus lenguas ancestrales, sobre cáncer de cuello uterino, sus factores de riesgo, la toma del examen y la forma de prevenirlo. Después de la intervención se realizó nuevamente la encuesta sobre conocimientos, actitudes y prácticas sobre cáncer de cuello uterino lo que permitió identificar la existencia o no de cambios de perspectiva sobre estos temas.

### Participación comunitaria

Las líderes femeninas, representantes de cada grupo étnico, fueron quienes participaron de forma activa en las etapas de la investigación-acción, con el apoyo de las autoridades del resguardo, quienes aprobaron los diferentes procesos y procedimientos propuestos para llevar a cabo la investigación.

(i) En la fase diagnóstica, participaron de manera activa cinco lideresas representantes de las etnias participantes, quienes realizaron las entrevistas, las tradujeron y transcribieron.

(ii) En la etapa relacionada con la construcción, puesta en marcha de la estrategia educativa y toma de la citología vaginal, participaron siete lideresas. Fueron seleccionadas por las autoridades indígenas por su capacidad de liderazgo, responsabilidad y manejo de los dos idiomas (español y lengua autóctona).

(iii) Las autoridades indígenas también participaron dando su apoyo, dedicando un espacio para invitar a las mujeres en sus reuniones semanales.

El seguimiento del trabajo fue realizado por dos investigadoras del proyecto, una médica y una antropóloga. Para el control de calidad se acompañó a las lideresas a realizar varias visitas. Además, también se organizaron reuniones periódicas para que las lideresas pudieran compartir sus experiencias. Una antropóloga realizó el acompañamiento permanente de las lideresas durante el trabajo de campo. La investigadora principal hizo visitas de campo cada 20 días por 8 a 10 días en promedio durante todo el desarrollo del proyecto. El trabajo se realizó en coordinación con los servicios de salud locales.

### Evaluación de la estrategia educativa

Para evaluar los resultados de la estrategia educativa se planteó aplicar una encuesta sobre conocimientos, actitudes y prácticas sobre el cáncer de cuello uterino antes y después de la intervención con la misma población. Sin embargo, no fue posible conservar el mismo grupo de mujeres para aplicar el cuestionario. Hubo dos motivos para esta limitación: (i) la alta movilidad de las mujeres del resguardo hacia los asentamientos ubicados a lo largo de los ríos que se adentran en la selva y (ii) no había censos poblacionales en las comunidades indígenas, por lo que no fue posible tener el marco muestral.

Para la indagación se propuso inicialmente el uso del formato validado de uso libre Cancer Awareness Measure versión 2 [*Medida de Concientización sobre el Cáncer*], traducido del inglés al español ^26^. Se realizó una prueba piloto, pero no resultó útil porque las lideresas no comprendían las preguntas, no podían traducirlas a las lenguas propias y tampoco eran claras para las mujeres entrevistadas.

Fue necesario hacer cambios importantes que derivaron en un formato diferente al inicialmente propuesto, acordando con las lideresas varios ajustes en cuanto a expresiones comprendidas por ellas y se procedió a realizar un cuestionario de preguntas abiertas y cerradas. La prueba piloto del nuevo formato arrojó un porcentaje de respuestas correctas antes de la intervención del 24% y se consideró que una prevalencia esperada de 40% de conocimientos aceptables después de la intervención podría tomarse como un cambio positivo de dicha actividad educativa. Para el cálculo del tamaño de muestra en la aplicación de la encuesta sobre conocimientos, actitudes y prácticas sobre cáncer de cuello uterino, se usó el módulo de *Comparación de Proporciones Emparejadas* de EPIDAT versión 4.3 (https://www.sergas.gal/Saude-publica/EPIDAT), estableciendo las proporciones del 24% y 40%, nivel de confianza del 95% y potencia estadística del 80% resultando un tamaño de muestra de 135 participantes, más el 10% por posibles pérdidas para obtener un tamaño de muestra final de 148 participantes.

Las respuestas de las preguntas abiertas fueron luego categorizadas y calificadas, lo que permitió realizar el análisis estadístico sobre las respuestas obtenidas. Para medir el nivel de conocimientos, se asignó un punto a cada pregunta si era correcta, para posteriormente sumar los puntos por cuestionario. Con un puntaje total máximo de 22 puntos y un mínimo aceptable igual o mayor a 11 puntos. Después se calculó el puntaje por temas: conocimientos sobre el cáncer de cuello uterino con un puntaje máximo de 13 puntos, sobre la citología vaginal con un puntaje máximo de 6 puntos y, sobre la vacuna contra el VPH, con un puntaje máximo de 3 puntos. Además, se calcularon los puntajes para cada una de las etnias participantes.

Los datos se ingresaron y codificaron en una base de datos en Microsoft Excel (https://products.office.com/) y en ella se realizó una depuración para corregir los posibles errores de digitación, posteriormente se exportaron a Stata versión 17 (https://www.stata.com) para el análisis. Se realizó una descripción general de las principales variables mediante un análisis univariado, para las variables categóricas se reportaron frecuencias absolutas y relativas y para las variables cuantitativas se reportaron mediante mediana y rango intercuartílico (RIC) de acuerdo con la evaluación previa del supuesto de normalidad con la prueba de Shapiro-Wilk.

Los efectos para todas las variables se determinaron con base en la comparación de cuestionarios resueltos antes y después de la intervención usando la prueba de Mann-Whitney para variables continuas y chi-cuadrado o prueba exacta de Fisher para las variables categóricas. Para todas las pruebas estadísticas se estableció un nivel de significancia de 0,05.

### Consideraciones éticas

El estudio fue aprobado por el Comité de Ética del Hospital de San José de Bogotá (Acta 9 de 24 de mayo de 2017). La participación de las mujeres fue voluntaria expresada mediante consentimiento informado que fue traducido a lenguas ancestrales. No se registraron datos de identificación en los cuestionarios para mantener la confidencialidad.

## Resultados

El total de mujeres que participaron en la estrategia educativa fue 957, con una participación mayor de los pueblos Puinave (337; 35,2%) Sikuani (285; 29,8%) y Piapoco (153; 16%). El rango de edad estuvo entre 13 a 93 años, con una mediana de 31 años (RIC: 23; 42).

En las jornadas de toma de citología vaginal, se logró una cobertura de 304 mujeres que incluyeron 3,29% de mujeres indígenas de otras etnias diferentes y 19% de mujeres mestizas quienes estuvieron interesadas en participar, pues no se negaron los servicios a ninguna mujer que asistió a solicitarlos.

La Tabla 1 muestra las características de las mujeres que contestaron la encuesta antes y después de la intervención. Los dos grupos fueron similares en cuanto a edad, nivel educativo, ocupación y número de hijos. La única variable que mostró diferencias estadísticamente significativas fue la composición por etnia.

**Tabla 1:** Características sociodemográficas de las mujeres que contestaron la encuesta antes y después de la intervención educativa.

Variable	Antes (n = 202)		Después (n = 176)		Valor de p
	n	%	n	%	
Edad [mediana (RIC)]	32 (24; 42)		32 (24; 41)		0,814
Nivel educativo					0,159
Ninguno	15	7,4	5	2,8	
Primaria	87	43,1	88	50,0	
Secundaria	89	44,1	75	42,6	
Técnico	11	5,4	7	4,0	
Ocupación					0,155
Ama de casa	153	75,7	135	76,7	
Empleada	23	11,4	15	8,5	
Estudiante	14	6,9	15	8,5	
Lideresa	3	1,5	0	0,0	
Trabajadora independiente	6	3,0	10	5,7	
Trabajadora de agricultura	3	1,5	0	0,0	
Desempleada	0	0,0	1	0,6	
Número de hijos [mediana (RIC)]	2 (1; 4)		2 (1; 4)		0,815
Pueblo/Grupo étnico					0,015 *
Cubeo	51	25,2	24	13,6	
Curripaco	19	9,4	17	9,7	
Piapoco	31	15,3	26	14,8	
Puinave	49	24,3	39	22,2	
Sikuani	52	25,7	70	39,8	

RIC: rango intercuartílico.

* Significativo al 5%.

Los datos presentados en la Tabla 2 muestran los conocimientos, actitudes y prácticas sobre el cáncer de cuello uterino de las mujeres antes y después de la intervención. Hubo un aumento estadísticamente significativo en la proporción de mujeres que han escuchado sobre el cáncer de cuello uterino, sobre la citología y sobre la vacuna contra el VPH. Igualmente, hubo cambios significativos en el conocimiento de los síntomas, sobre algunos de los factores de riesgo como no hacerse el examen de citología y reconocer su importancia para la prevención de enfermedades.

**Tabla 2:** Respuestas sobre el cáncer de cuello uterino antes y después de la intervención educativa.

Pregunta	Antes		Después		Valor de p
	n	%	n	%	
¿Usted ha oído hablar del cáncer de cuello uterino?					
Sí	125	62,0	168	95,0	< 0,0001
No	77	38,0	8	5,0	
¿Qué siente una mujer que tiene cáncer de cuello uterino?					
Secreción vaginal sanguinolenta o fétida	9	4,1	33	18,8	< 0,0001
Dolor abdominal, pélvico o lumbar	52	27,7	103	58,5	< 0,0001
Sangrado intermenstrual o postmenopáusico	41	20,3	61	34,7	< 0,0001
Pérdida de peso sin causa explicable	4	2,0	2	1,1	0,4841
Hematuria o deposición con sangre	1	0,5	1	0,6	0,8953
No sabe	116	57,4	23	13,1	< 0,0001
¿Si tuviera alguno de estos síntomas usted consultaría a alguien?					
Sí	114	91,0	161	97,0	< 0,0001
No	11	9,0	5	3,0	
¿A quién consultaría?					
Médico tradicional	9	8,0	7	4,0	
Servicio de salud occidental	97	84,0	139	86,0	0,7292
Ambos	9	8,0	16	10,0	0,5479
¿Por qué se enferman las mujeres de cáncer de cuello uterino?					
Tener el VPH	3	1,5	0	0,0	0,1028
No hacerse la citología	19	9,4	48	27,3	< 0,0001
Multiparidad	9	4,5	9	5,1	0,785
Inicio de relaciones sexuales antes de los 17 años	6	3,0	1	0,6	0,0868
Tener varios compañeros sexuales	21	10,4	44	25,0	0,0002
Tabaquismo	2	1,0	1	0,6	0,6659
Uso prolongado de anticonceptivos	6	3,0	0	0,0	0,0205
No sabe	135	66,8	38	38,6	< 0,0001
¿Usted ha oído hablar de la citología vaginal?					
Sí	169	83,7	169	96,0	< 0,0001
No	33	16,3	7	4,0	
¿Para qué se hace la citología vaginal?					
Detectar algún problema o enfermedad en el cuello de útero o saber si uno tiene cáncer	37	18,3	35	19,9	0,6983
Detectar algún problema o enfermedad	97	48,0	109	61,9	0,0067
No sabe	68	33,7	32	18,2	0,0007
¿Alguna vez le han hecho citología vaginal?					
Sí	130	64,4	130	73,9	0,0467
No/No recuerda/No sabe	72	35,6	46	26,1	0,0467
¿Reclamó los resultados?					
Sí	92	70,8	91	70,0	0,8876
No/No recuerda/No sabe	38	29,2	39	30,0	0,8876
¿Hace cuánto le hicieron la última citología?					
Menos de tres años	76	37,6	81	46,0	0,0983
Más de tres años	55	27,2	48	27,3	0,9826
No se la ha realizado	71	35,2	47	26,7	0,0753
¿En el hospital o en el centro de salud toman las citologías?					
Sí	159	94,1	155	91,7	0,3969
No o no sabe o no responde	10	5,9	14	8,3	0,3969
¿Desde cuándo las mujeres deben comenzar a hacerse la citología?					
Después de comenzar la vida sexual	43	25,4	73	43,2	0,0006
No sabe/No responde	126	74,6	96	56,8	0,0006
¿Ha oído hablar de la vacuna contra el VPH?					
Sí	140	70,4	158	90,8	< 0,0001
No	59	29,6	17	9,2	
¿A qué edad se deben vacunar las niñas?					
Entre 9 y 14 años	107	76,4	139	88,0	0,0088
No sabe/No responde	33	23,6	19	12,0	0,0088
¿Usted vacunaría a sus hijas contra el VPH?					
Sí	113	95,8	132	97,1	0,5773
No	5	4,2	4	2,9	0,5773

VPH: virus del papiloma humano.

Llama la atención que el escaso conocimiento que tenían sobre la relación del VPH con el cáncer de cuello uterino, no cambió con la intervención. Tampoco cambiaron los conocimientos sobre factores de riesgo importantes como la multiparidad o el inicio temprano de relaciones sexuales, temas que fueron tratados en las charlas educativas y consignados en los materiales escritos.

Es de resaltar la alta proporción de mujeres con una actitud positiva hacia la consulta al médico occidental antes y después de la intervención educativa (84% y 86% respectivamente). Se observa también una actitud positiva hacia vacunar a las hijas contra el VPH (96% antes de la intervención y 97% después). Además, se observa una alta proporción de mujeres que tienen conocimiento de que en el hospital toman citologías (95% antes de la intervención y 96% después) (Tabla 2).

Adicionalmente, se observó un aumento estadísticamente significativo respecto a la práctica de la citología (64,4% antes y 73,9% después de la intervención; p = 0,0467). Sin embargo, no se observaron cambios en cuanto a la práctica de reclamar los resultados del examen (Tabla 2).

En relación al nivel de conocimientos sobre el cáncer de cuello uterino, se encontró un aumento estadísticamente significativo en el puntaje total de la población encuestada y en el de los pueblos Puinave, Sikuani y Piapoco con una mediana de 13, 11,8 y 12,5 respectivamente. Sin embargo, el nivel de conocimientos después de la intervención fue mínimo aceptable (12,5%; RIC: 10,5; 13,5), teniendo en cuenta que el puntaje máximo era de 22 puntos.

**Figura 1:**
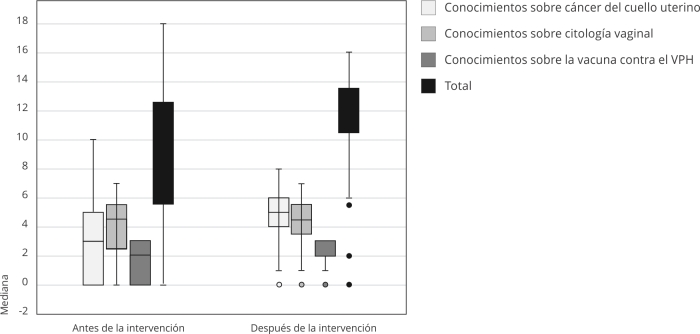
Mediana del puntaje antes y después de la intervención educativa respecto a los conocimientos sobre el cáncer del cuello uterino.

En la Figura 1 se compara la mediana del puntaje total y el de algunos temas específicos observándose un incremento significativo en los puntajes de los conocimientos respecto al cáncer de cuello uterino aumentó de 3,0 a 5,0 (p ˂ 0,0001) y la vacuna contra el VPH de 2,0 a 3,0 (p ˂ 0,0001) después de la intervención, mientras que la mediana del puntaje del tema de citología vaginal se mantuvo.

En la Tabla 3 se presentan los cambios en el conocimiento antes y después de la intervención sobre tres aspectos mínimos de sensibilización sobre el tema: haber oído hablar del cáncer de cuello uterino; haber oído hablar de la citología vaginal y haber oído hablar de la vacuna contra el VPH. Estos cambios se exploraron en relación con el nivel educativo.

**Tabla 3:** Relación entre el nivel educativo y los conocimientos sobre el cáncer del cuello uterino antes y después de la intervención educativa.

Conocimientos/Nivel educativo	Antes		Después		Valor de p
	n	%	n	%	
¿Usted ha oído hablar del cáncer de cuello uterino?					
Ninguno					
Sí	3	20,0	3	60,0	0,091
No	12	80,0	2	40,0	
Primaria					
Sí	47	52,2	86	97,7	< 0,0001
No	43	47,8	2	2,3	
Secundaria					
Sí	64	74,4	72	96,0	0,0002
No	22	25,6	3	4,0	
Técnico					
Sí	11	100,0	7	100,0	
No	0	0,0	0	0,0	
¿Usted ha oído hablar de la citología vaginal?					
Ninguno					
Sí	6	40,0	3	60,0	0,4363
No	9	60,0	2	40,0	
Primaria					
Sí	75	83,3	87	98,9	0,0003
No	15	16,7	1	1,1	
Secundaria					
Sí	77	89,5	72	96,0	0,1177
No	9	10,5	3	4,0	
Técnico					
Sí	11	100,0	7	100,0	
No	0	0,0	0	0,0	
¿Usted ha oído hablar de la vacuna contra el VPH?					
Ninguno					
Sí	4	28,6	3	60,0	0,2116
No	10	71,4	2	40,0	
Primaria					
Sí	56	62,9	77	88,5	0,0001
No	33	37,1	10	11,5	
Secundaria					
Sí	69	81,2	71	94,7	0,0099
No	16	18,8	4	5,3	
Técnico					
Sí	11	100,0	7	100,0	
No	0	0,0	0	0,0	

VPH: virus del papiloma humano.

Respecto al cáncer de cuello uterino, se observaron cambios en el grupo de nivel educativo primario y secundario, con un aumento notorio en la proporción de mujeres que respondieron afirmativamente después de la intervención, alcanzando valores cercanos al 100%. Este cambio también fue importante en el grupo de mujeres sin ningún nivel educativo, aunque no fue estadísticamente significativo.

En relación con la citología, los tres niveles educativos inferiores, mostraron un incremento después de la intervención en la proporción de mujeres con respuesta afirmativa, aunque la diferencia fue estadísticamente significativa únicamente para el grupo de educación primaria.

Respecto a la vacuna contra el VPH, hubo cambios positivos en los tres niveles inferiores, aunque fueron estadísticamente significativos solamente para los niveles primario y secundario.

## Discusión

Este estudio realizado desde las lógicas de la IAP, aporta elementos importantes para la comprensión de la importancia del desarrollo de actividades de promoción de la salud y prevención de la enfermedad desde ópticas interculturales y a partir de los protagonistas de los procesos. El trabajo con las lideresas permitió, no solo que ellas mismas realizaran un proceso de aprendizaje sobre el cáncer de cuello uterino, su prevención y tratamiento, sino que se convirtió en la posibilidad de generar empoderamiento sobre la salud de sus propias comunidades, pues como pares de las mujeres de sus etnias, estas influyen en procesos de transformación que mejoran el acceso a los servicios de salud ^17^ ^,^ ^19^ ^,^ ^27^ ^,^ ^28^.

En relación con los efectos de la intervención educativa, se resalta que esta aporta no solo resultados cuantitativos estadísticamente significativos, incluido un mayor conocimiento sobre el cáncer de cuello uterino, las pruebas de detección y la vacunación contra el VPH, sino que demuestra que el trabajo en salud con y para las comunidades, puede conciliar los conocimientos culturales propios con los científicamente aceptados. Lo anterior se evidenció con la asistencia de las mujeres indígenas a las jornadas de toma de citologías realizadas durante el estudio y la participación voluntaria en las actividades que se realizaron para implementar la estrategia educativa. Situación que se corresponde a los estudios relacionados con la eficacia de intervenciones para la prevención del cáncer de cuello uterino, donde las estrategias educativas aportan de manera importante en este aspecto ^29^ ^,^ ^30^ ^,^ ^31^ ^,^ ^32^ ^,^ ^33^ ^,^ ^34^.

Uno de los aspectos que llama la atención es que la mayoría de las mujeres indígenas ya conocían alguna información sobre el cáncer de cuello uterino. Sin embargo, un 35,6% nunca se habían practicado la citología vaginal, cifra superior a la reportada en otros estudios en la Región Amazónica de Colombia ^35^, lo que resalta la importancia de los programas de prevención y detección precoz del cáncer de cuello uterino en estas comunidades. Además, no es suficiente promover la práctica del tamizaje si no se realiza el proceso completo de lectura de resultados, pues como se vio en el estudio, algunas mujeres se toman la citología, pero no reclaman el resultado, lo que se corresponde con otros estudios ^36^ ^,^ ^37^ ^,^ ^38^.

Un punto a resaltar es la importancia de las estrategias de información y educación por pares, porque uno de los factores que influyeron en la respuesta a la convocatoria para la realización de la actividad educativa y toma de citología, fue el contacto de las mujeres con mujeres de su propia etnia, pues esto genera confianza y mejor aceptación de las actividades propuestas por el sector salud, estrategia que ha venido siendo reportada como exitosa en otros trabajos relacionados con prevención y tratamiento de enfermedades ^39^ ^,^ ^40^. Es de resaltar el papel de las lideresas indígenas, quienes se convirtieron en facilitadoras del proceso y además ganaron en autonomía, liderazgo y reconocimiento por las autoridades dentro de sus comunidades. Lo anterior contribuye a reducir las inequidades de género que han sido descriptas en las mujeres indígenas que sufren de un cúmulo de negación de derechos y una doble condición de invisibilidad por su identidad indígena y por ser mujer ^41^. En general, la recepción de la estrategia educativa fue buena, sin embargo, dentro de las dificultades presentadas, algunas mujeres indígenas no quisieron participar en las entrevistas ni en la toma de citologías, especialmente de la etnia Sikuani.

No obstante, a pesar del aumento del conocimiento sobre el cáncer de cuello uterino, se observó que el nivel de conocimientos fue un mínimo aceptable. Algunos estudios han evidenciado pequeños efectos de las intervenciones educativas sobre la detección del cáncer contrario a otras revisiones que han mostrado mejores resultados ^42^ ^,^ ^43^. Respecto a los aspectos que contribuyen al éxito de estos programas, se han identificado la adaptación cultural, los componentes individualizados, los múltiples contactos, la retroalimentación e involucrar educadores comunitarios ^16^ ^,^ ^21^ ^,^ ^43^, aspectos que fueron incluidos en esta investigación.

De acuerdo a lo anterior, en el contexto del proyecto realizado, se considera que los aspectos que limitaron el éxito de la intervención educativa son un complejo de circunstancias como la falta de confianza de las comunidades derivada de las tensiones raciales históricas y actuales, las diferencias en las lógicas y tiempos de las comunidades indígenas con los occidentales, los periodos de financiación de los proyectos poco realistas que dificultan la consolidación de las acciones y relaciones, y la falta de comprensión de las comunidades sobre la importancia de la educación para la salud porque no perciben el cáncer de cuello uterino como un problema. Además, como se describió en la metodología, la encuesta se aplicó a muestras independientes en el pretest y postest, debido a la alta movilidad de las comunidades del resguardo hacia otros resguardos, hacia las fincas y los ríos durante semanas o meses.

No obstante, a pesar de que el aumento en los conocimientos sobre el cáncer de cuello uterino fue un mínimo aceptable, es de resaltar que este aumento después de la intervención se dio principalmente en las mujeres indígenas de menor nivel educativo. Resultados que corroboran que el nivel educativo está asociado con los conocimientos sobre cáncer de cuello uterino como uno de los determinantes sociales ^44^ ^,^ ^45^, lo que justifica la focalización de las intervenciones educativas en los niveles socioeconómicos más bajos para disminuir las brechas en salud. Teniendo en cuenta que la Amazonía colombiana presenta tasas elevadas de cáncer de cuello uterino en relación con el resto del país ^46^, trabajos como este disminuyen las inequidades en poblaciones indígenas, quienes se han visto limitadas para tener acceso a los servicios de salud oportunos debido a su localización geográfica, la desconfianza con las instituciones, la falta de conocimientos y la ausencia de atención integral.

Esta investigación aporta elementos importantes para el diseño de estrategias para la promoción de la salud fundamentados en la IAP. Tal como lo plantea Santoro ^47^, en este trabajo se hizo evidente que los procesos participativos son útiles para adaptar las políticas globales a entornos específicos, logrando mayor impacto de los programas de salud. La participación es un primer paso para la toma de consciencia de los problemas de salud que abrirá el camino a etapas más avanzadas enfocadas a la transformación de la realidad y el empoderamiento de los grupos indígenas. Se recomienda que las entidades financiadoras de estos proyectos, promuevan la ejecución de proyectos a largo plazo que permitan desarrollar mejores procesos y de mayor duración en estas comunidades para avanzar hacia dichas etapas y crear relaciones horizontales con las instituciones de salud.
